# “There's not enough studies”: Views of black breast and ovarian cancer patients on research participation

**DOI:** 10.1002/cam4.5622

**Published:** 2023-01-16

**Authors:** Kirsten A. Riggan, Abigail Rousseau, Michele Halyard, Sarah E. James, Marion Kelly, Daphne Phillips, Megan A. Allyse

**Affiliations:** ^1^ Biomedical Ethics Research Program Mayo Clinic Rochester Minnesota USA; ^2^ Department of Radiation Oncology Mayo Clinic Phoenix Arizona USA; ^3^ Coalition of Blacks Against Breast Cancer Phoenix Arizona USA; ^4^ ADVANCE Community Advisory Board Phoenix Arizona USA; ^5^ Department of Community Engagement Mayo Clinic Scottsdale Arizona USA; ^6^ Department of Speech Pathology Mayo Clinic Phoenix Arizona USA; ^7^ Department of Obstetrics & Gynecology Mayo Clinic Rochester Minnesota USA

**Keywords:** breast cancer, clinical cancer research, community outreach, women's cancer

## Abstract

**Background:**

Black breast and ovarian cancer patients are underrepresented in clinical cancer trials disproportionate to the prevalence of these cancers in Black females. Historically, lower enrollment has been attributed to individualized factors, including medical mistrust, but more recently structural factors, including systemic racism, have received additional scrutiny. We interviewed Black women with a personal or family history of breast and ovarian cancer to understand their views and experiences related to research participation.

**Methods:**

Qualitative interviews were conducted via telephone or video conference and transcribed verbatim. Transcripts were qualitatively analyzed for iterative themes related to the offer and participation in cancer clinical trials and research studies, impact on cancer care, and recommendations to increase enrollment of Black patients.

**Results:**

Sixty‐one Black women completed an interview. Participants expressed that Black women are underrepresented in cancer research, and that this negatively impacted their own care. Many cited past historical abuses, including the Tuskegee syphilis trial, as a potential factor for lower enrollment but suggested that lower enrollment was better understood in the context of the entirety of their healthcare experiences, including present‐day examples of patient mistreatment or dismissal. Participants suggested that proactive community engagement, transparency, and increased representation of Black research team members were strategies likely to foster trust and bolster research participation.

**Conclusion(s):**

Medical mistrust is only a partial factor in the lower participation of Black patients in cancer research. Researchers should implement the strategies identified by our participants to promote diverse enrollment and ensure that Black patients are included in future therapeutic advances.

## 1 INTRODUCTION

There are stark racial disparities in mortality from breast and ovarian cancer in the United States (U.S.). Despite similar or lower incidence rates, non‐Hispanic Black/African American (“Black”) patients have a 41% higher mortality rate for breast cancer and experience the lowest survival for ovarian cancer across most subtypes compared with non‐Hispanic White patients.^1^, ^2^ These disparities are multifactorial—including later stage of diagnosis, treatment delays, non‐guideline‐adherent care and the enrichment of aggressive cancer subtypes. There is also the categorical influence of socioeconomic status, environment/geographic constraints, social determinants of health, and structural racism.^3^, ^4^, ^5^, ^6^, ^7^, ^8^, ^9^

One key predictor of survivorship is access to innovative care via clinical trials. However, enrollment of Black patients is disproportionate to the U.S. Black population and cancer prevalence. A retrospective review found that only 7.2% of Black breast cancer patients were enrolled in a clinical trial, among the one‐third of trials that reported ethnicity.^10^ A 32‐fold lower enrollment is observed for Black women with ovarian cancer.^11^ Thus, current treatment standards have been largely formulated by data with the near exclusion of Black women.^12^ The lack of reporting of race/ethnicity and low inclusion of non‐White groups in clinical trials and epidemiology studies have contributed to the poor understanding of ethnicity‐based differences in treatment response, including biological and psychosocial factors that potentially influence disparate outcomes.^13^, ^14^

Low enrollment of racialized groups in clinical trials is typically attributed to a lack of trust in the medical enterprise and minimal patient education. Recently, scholars have pushed back against this individualistic characterization, centering on structural and socioeconomic factors that frequently exclude Black individuals from inclusion. In assessing the significant gap in Black enrollment in clinical trials in breast and ovarian cancer, we determined it was important to speak directly to women who had experiences with cancer or had a first‐degree relative with a history. As part of a larger qualitative interview study of Black women, we explored their perceptions and experiences on cancer research participation and their recommendations for the inclusion of Black participants in clinical research.

## 2 METHODS

### 2.1 Community engagement

We partnered with the Coalition of Blacks Against Breast Cancer (CBBC), a nonprofit organization offering cancer education and support for Black women and their families, to form a community advisory board (CAB) composed of 8‐12 members with a personal or family history of breast and ovarian cancer.^15^ The CAB also included allied health professionals and community advocates. The CAB advised on all aspects of the study, including recruitment material, interview guides, and participant remuneration. Members also assisted with recruitment and provided feedback on preliminary results.

### 2.2 Recruitment

The study was approved by Mayo Clinic's Institutional Review Board as minimal risk. Inclusion criteria included female and Black or African American, and a personal diagnosis or biologically related to a family member diagnosed with breast or ovarian cancer. Participants were recruited through the social media platforms and email listservs of patient support organizations including CBBC, the National Ovarian Cancer Coalition, the Sister's Network of Southeast Florida, Black sororities and professional organizations, and through snowball recruitment. Patients meeting inclusion criteria from Mayo Clinic Jacksonville's Cancer Center were also invited to participate. Due to social media recruitment and the use of snowball sampling, a response rate cannot be determined. Following completion of an online screening survey or verification of eligibility by the study team, participants were invited to schedule a phone or video conference interview. All participants provided written consent and HIPAA authorization. Following interview completion, participants received a gift basket of items as thanks for their participation.

### 2.3 Data collection

One researcher (KR) with experience in breast cancer and qualitative research conducted semi‐structured interviews using two interview guides for participants with a personal diagnosis or family history of breast or ovarian cancer. The interview guides were developed by members of the study team with expertise in oncology, patient experience, community engagement, bioethics, and qualitative methods. The interview guides were designed to explore a broad spectrum of participants' cancer experience, including their diagnosis, family history, medical experiences, cancer support, genetic testing, and research participation. Interviews were conducted until thematic saturation was reached and were audio recorded with permission (n = 60) and transcribed verbatim. The average interview length was 82 minutes (range 43‐152 min) for participants with a personal diagnosis and 62 minutes (range 42‐89 min) for those with a family history.

### 2.4 Data analysis

Two researchers (KR and KC) developed separate codebooks for each cohort.^16^ Four (9.3%) randomly selected transcripts were used to develop the codebook for those with a personal diagnosis and 2 (11.1%) from family members. Transcripts were divided and coded using the software program NVivo12 between three researchers (KR, n = 24, 39.3%; AR, n = 16, 26.2%; KC, n = 8, 13.1%). At least two researchers (KR and KC, KR and AR) coded an additional 13 transcripts (21.3%) to consensus at periodic intervals for coding consistency and accuracy. The research team met weekly to discuss preliminary themes and resolve coding discrepancies. In accordance with the standards of reporting qualitative research,^17^ representative quotes are provided with minimal editing for readability. Here, we present the thematic analysis of content coded to research experience codes, which includes content on personal interest, perceived benefits, and concerns about research participation, specific factors that may influence participation of Black patients, and recommendations for improving recruitment and retention of Black participants. A sample coding schema is available in the Supplemental Materials (Table S1). All coded content that pertained to research participation was inductively analyzed by one researcher (KR) to identify emerging themes; findings were discussed with the larger research team for feedback and agreement.

## 3 RESULTS

Of the 89 individuals who met the inclusion criteria, 66 (74.2%) returned their consent form, and 61 (68.5%) completed an interview. Forty‐three participants had a personal diagnosis (70.5%) and 18 (29.5%) had a family member with a diagnosis of breast or ovarian cancer. Eight participants were mother‐daughter dyads. Table 1 describes the demographics of the sample.

**TABLE 1:** Demographics

Characteristic	Personal Diagnosis (N = 43)	Family member diagnosed (N = 18)
Gender		
Female	43 (100%)	18 (100%)
Cancer		
Breast	37 (86.0)	14 (77.8)
Ovarian	6 (14.0)	2 (11.1)
Breast and ovarian	0 (0.0)	2 (11.1)
Mean age (Range)	59.5 (31‐86) yr.	46.3 (29‐66) yr.
Education		
High school	1 (2.4)	1 (5.6)
Some college	5 (11.9)	1 (5.6)
Associate degree	5 (11.9)	0 (0.0)
Bachelor's degree	7 (16.7)	4 (22.2)
Some graduate	1 (2.4)	2 (11.1)
Graduate degree	24 (57.1)	10 (55.6)
Health status		
Fair	13 (30.2)	3 (16.7)
Good	21 (48.8)	13 (72.2)
Excellent	9 (20.9)	2 (11.1)
Insurance		
Employer or Private	30 (69.8)	
Public	12 (27.9)	
Other/Not reported	1 (2.3)	

### 3.1 Participation in cancer clinical trials and research studies

Many participants expressed that breast and ovarian cancer research is underdeveloped for Black patients and families. In some cases, they felt that the lack of ethnicity‐specific data on cancer therapeutics and treatment options negatively impacted their own care.

I would ask my healthcare provider about it [treatment option], and she would go, “No, there's not enough studies.” (…) I just wanted to make certain I was included in the greatest and the latest thing that was happening. I never felt that, never. I just felt like I was being given what the status quo—these are the three medications. A4 (Age 55, Breast Cancer Diagnosis)

Awareness of this lack of data often served as a motivating factor to participate in research, the present study included, and even consider more invasive clinical trials.

African American women and metastatic breast cancer are two areas that really, really need more funding and more research. It's important that for me, that they get as much—get more participants. That's why when I do see studies that I can participate in, I will participate in them. A9 (Age 37, Breast Cancer Diagnosis)

Seventeen participants with a personal diagnosis indicated that they were offered participation in a clinical trial or research study related to their cancer care. Of those, 12 agreed to participate. Of those that did not receive an offer, several believed it was because there was an established treatment regimen for their cancer subtype and research was not needed. However, some participants expressed dismay that they were not offered research participation.

I do not believe a lotta African Americans are involved in studies as such or clinical trials because throughout my research, I really did not find much. It would've been nice to actually been offered or be able to participate or even to consider it, but it did not seem like it was an option that was even presented to me. A22 (Age 50, Breast Cancer Diagnosis)

A few who were offered, but declined, participation in a clinical trial or research study indicated that they did so because of concerns related to past historical research abuses.

I declined [clinical trial]. The reason I did was because of our history with research projects. All I knew at that time was the bad news of the Tuskegee and other experiments…As much as I would do anything to help at that time, I had no faith. Although I worked in health care for, at that point, almost 20 years, I just had no faith in the system itself. A13 (Age 59, Breast Cancer Diagnosis)

Many participants felt increased participation of Black individuals would improve treatment options and outcomes for Black women with breast and ovarian cancer.

I know a lot of research and medicine that is recommended, the dosage and all of that is based on white males. Our bodies, our geneology, our everything is so different from them. We need more women, more minorities to participate in research so that we can better treat different diseases. A45 (Age 60, Breast Cancer Diagnosis)

A few participants who were especially interested in participating in a research study stated that they were searching specifically for opportunities related to long‐term recovery as a means to support their physical and mental health needs and maintain continuity of care.

Everything is over. It was just like radio silence. Thankfully, a month and a half later, I started the [name] study, or otherwise, I probably would've gone into a really deep depression because there was just so much had happened. A4 (Age 55, Breast Cancer Diagnosis)

### 3.2 Barriers to participating in research studies

Thirty‐five participants made specific or general reference to past historical abuses (e.g., Tuskegee syphilis study; Henrietta Lacks; involuntary sterilization) as a potential reason why Black individuals may be less likely to participate in research. They recounted a continued perception among Black patients that researchers have malevolent intentions or that Black individuals are used as “guinea pigs” rather than being viewed as equal and valued participants in research.

I think that Tuskegee study is still in our minds of many of us, and we wonder, if it's gonna benefit me, or are they gonna get—collect this information, and they are not going to treat me? They're going to just get the information, and I think I'm being treated, but no, they are giving a sugar pill, and they are watching something advance in my body to help somebody else? I think there is a reluctance in our community to participate in those kinds of trials. A20 (Age 76, Breast Cancer Diagnosis)

Some felt that these historical concerns were less prevalent among younger generations and that generational attitudes were shifting.

The first thing that comes up was, “Well, remember what they did with black people with syphilis. Who wants to go do that again?” There's a historical conversation that comes up immediately (…) I think younger people are more likely to say, “Sure, why not? I'd like to try.”. A12 (Age 64, Sister Diagnosed with Breast Cancer)

A few participants suggested that the Tuskegee syphilis study has been mythologized or that misinformation is common about the study. Other cited specific fears or conspiracies on scientific research that they have heard circulated in their communities.

I think it's the whole conspiracy theory of they are gonna take your genetic information and they are gonna be using it to try to—I do not know, to clone you, or put it in the system. Put it in some kind of database, and that kind of ignorant stuff. I do not want people diggin’ around in my DNA, or asking me questions. It's a trust thing, the trust. A3 (Age 48, Breast Cancer Diagnosis)

Others, however, felt that lower participation in research among Black individuals could not be reduced to issues of historical mistrust of the research establishment. These participants identified other factors they felt were more likely behind the disparity, including present‐day examples of medical and research misconduct.

There's just a lack of trust. You like to think that we are past that kind of atrocity being perpetrated against humans, but then we find out yesterday that they are removing the uteruses of women in detention, and so you cannot trust. It's unfortunate. A19 (Age 59, Mother Diagnosed with Breast Cancer)

Participants described their own or family and friends' experiences receiving medical care, including examples of racial discrimination, weight bias, perceptions that their pain and symptoms were dismissed, lack of personal investment of providers in their care, and systemic racism as potential sources of hesitation to participate in research studies.

From the questions you asked about “how has your experience been with your current provider or with other providers?” (…) I think those kindsa questions answers the question of whether other black folk or whether black people in general [go] into clinical trials. A23 (Age 62, Breast Cancer Diagnosis)

### 3.3 Participant recommendations to improve research participation

Participants offered several recommendations on how to improve research participation among Black individuals. First, they felt researchers should improve their education and outreach efforts. This includes engagement with Black community members early in the research process and clearly explaining the potential benefits of research participation to participants and their community.

I feel, if we were given the information to look it over and look through it—we were made a part of the decision to actually be a part of the study, I think other African Americans would do it or would wanna be a part of it, as long as they feel like it's gonna make a difference, and what is it about? Let me find out what it's about. I think goin’ in blindly, they probably will not. A51 (Age 44, Breast Cancer Diagnosis).

Some participants felt that the oft‐cited issue of medical mistrust could be easily addressed with greater community engagement.

Because people talk about mistrust in the African American communities, I do not think that's as big of a deal as most people think. This is just myself. I think when people are educated about clinical trials, about you have the potential for the latest and greatest, and possibly could extend your life, and also your impact on humankind of the information that you are giving. I think given the right information that mistrust can be melted in a second. I've seen it happen. A47 (Age 61, Breast Cancer Diagnosis)

Participants also suggested that increased representation of Black physicians and researchers both among the research team and medical community more broadly will reassure Black participants that their interests and concerns will be advocated for. Others suggested that study information and the offer of participation should come from a trusted member of the local community.

Probably also because there's not enough representation of, in my case, Black medical physicians that may be a part of these studies that could provide the insight necessary to make them more—more effective. I think, again, that if I was asked to be in a research study, but yet it was quote “endorsed by” medical professionals like myself, whether it's African American woman or an African American in general, that might cause me to be more inclined because I would feel that there was somewhat oversight or some knowledge that this is a quote “legitimate” study. A32 (Age 66, Mother and Aunt Diagnosed with Breast Cancer)

Some participants were motivated to serve as research advocates in their communities and described their own efforts towards breaking stigmas around research participation.

I keep telling everybody that talks about this, if you want to help people to get past it [historical abuses], you need to have brown people talking to them about it. You need to have people who look like them that they trust to say, “Hey, it's okay.” I keep telling every person I know, “I'm in a study, and it's not bad. It's not a problem.” It's just, keep cycling that message over and over again. A25 (Age 58, Mom and Sister Diagnosed with Breast Cancer)

Improving access to quality medical care was also thought to lower practical barriers to participation. These participants felt that increased access and engagement with quality medical care will, in turn, normalize the offer of research participation.

I was thinkin’ about how they always say they cannot reach enough Black women with breast cancer. Why do not you put a clinic over there in the center of the Black community? [Hospital] is way over here on the other side [of] town, nowhere near anybody. They put a clinic right in the middle, over there, wherever the Black community is. Make the community aware of it. A1 (Age 55, Breast Cancer Diagnosis)

Participants also stressed that it is important that researchers and institutions be open and transparent about past historical abuses in their present efforts to engage Black communities. The same participant who declined participation because of this shared past, described how community outreach has changed her perception of clinical research:

The fact that researchers, the research industry recognized the disparity which occurred in times past is the only reason I even consider it [research participation] now (…) I hear you guys reaching out (…) I hear you doing it. I hear it in my church. I hear it, and so I appreciate the diligence because that's the only way we'll know how things impact our group, us as a group. A13 (Age 59, Breast Cancer Diagnosis)

## 4 DISCUSSION

Despite repeated calls for the inclusion of ethnically diverse participants, Black patients continue to be underrepresented in cancer research.^13^, ^14^, ^18^ In 2020, the U.S. Congress mandated that the National Institutes of Health fund a National Academies of Sciences, Engineering, and Medicine Committee to study the long‐term medical and economic effects of the lack of inclusion of racial/ethnic minorities in medical research. In addition to the lack of generalizability of research findings and significant economic gains associated with improved representation, the task force concluded underrepresentation perpetuates mistrust in medicine and compounds existing health disparities.^19^ Historically, individual‐level factors have been cited to explain the low participation of Black individuals in clinical research, most notably feelings of mistrust originating in past scientific misconduct, including, but not limited to, the Tuskegee Syphilis Study.^20^, ^21^ Low socioeconomic status, health literacy, and educational attainment have also been cited as reasons for poor recruitment.^22^, ^23^, ^24^, ^25^, ^26^ Other studies, however, have questioned the validity of this conclusion, finding that Black individuals and other racial/ethnic groups are no less likely to participate in research as compared to other groups.^27^, ^28^

Participants attributed the lack of data for breast and ovarian cancer therapeutics in Black patients to a lack of research interest in Black health and survival. Nevertheless, many expressed hope for partnership between the medical establishment and Black communities, including the prioritization of community‐specific values in service of the larger goal of mitigating existing breast and ovarian cancer disparities. Similar to previous reports,^21^ the legacy of the Tuskegee Syphilis Study and other high‐profile historical examples of exploitation and abuse, were cited as sources of tempered interest in research participation and a cause for significant mistrust. Other participants discussed personal examples of racial bias and discrimination encountered in medical settings, including feelings that their medical concerns, and those of Black patients more generally, are frequently dismissed by healthcare providers. A lack of respect in clinical care reinforced perceptions of differential treatment by the medical establishment. Black research participation should be considered in the context of the totality of their healthcare experiences, including the intersection of gender and racial biases and negative patient‐provider encounters.^29^, ^30^

Multiple participants cautioned against a simplified association of medical mistrust and lower Black research participation, and in particular, over‐reliance on the touchstone of the Tuskegee Study. They suggested that research participation is nuanced, multifactorial, and includes a broader range of social factors than race/ethnicity. Other factors included insufficient time to complete research activities and the perception that cancer trials are only for patients for whom all other treatment options have been exhausted. The concept that clinical trials are a “last resort” is commonly reported in the qualitative literature on research participation.^31^ Participants indicated that structural barriers, such as less access to tertiary medical centers and poor healthcare quality, were the primary drivers for low awareness and participation. There is emerging evidence supporting this: Multiple studies have demonstrated that Black individuals are no less likely to participate in cancer research when given the opportunity.^27^, ^28^, ^32^, ^33^

Many participants provided suggestions to improve the recruitment and retention of Black participants. Most comments centered on improving community information and removing structural barriers. They strongly endorsed greater community engagement, with an emphasis on engaging with religious organizations, and physically centering research activities in Black communities. Lack of study awareness, including limited clinician discussion of available trials, is cited as one of the primary barriers to research participation among minority communities.^26^, ^34^, ^35^ Participants also emphasized the importance of representation; some reported that they would be more likely to participate in research if Black researchers were part of the research team, similar to other reports.^36^ The importance of community inclusion early in study development was also stressed as a means to build trust and ensure that the health of Black people is a research priority. Community‐engaged research is an effective means of increasing study enrollment, suggesting that the continued expansion of these efforts will have bidirectional dividends for both researchers and underserved Black communities.^37^, ^38^, ^39^

### 4.1 Designing research to expand access

Clinicians serve as important gatekeepers to patient awareness of and recruitment to clinical trials. A recent interview study examining the role of bias and stereotyping in research recruitment of minorities for oncology clinical trials found that some research professionals and staff viewed potential minority patients as unsuitable candidates for research due to likely noncompliance with research protocols, additional time needed to explain the clinical trial, and generally were perceived to be more difficult to interact with.^40^ These findings are supported by a prior small mixed methods study of oncology patients, which found that there was less discussion of clinical trials with African American compared to White patients.^41^ Our own findings are also consistent with this literature; many participants were dismayed that the option to participate in a research study or clinical trial was not discussed by their cancer care team. A separate analysis of the same research professionals highlighted the desire for specific cultural awareness and recruitment training for minority participants.^42^ Together, this suggests a continued need to address implicit and explicit racial bias at the clinician level to improve the recruitment of diverse patients.

Incorporating community providers into the research enterprise is another strategy that has been identified to improve diverse enrollment.^43^ This approach may be particularly beneficial for research involving previvors and long‐term recovery. Several participants indicated that they would be particularly interested in studies examining the long‐term physical and mental effects of their cancer diagnosis and treatment in the context of their postcancer care needs. Trust in physicians is also a repeated theme in qualitative studies examining factors influencing research participation, suggesting that the offer of study participation may best come from a provider with whom the patient has a long‐term relationship and rapport.^31^

More broadly, there is a role for all research professionals and staff to advocate for institutional policies and procedures that are inclusive of diverse participants. This may include ensuring that barriers to study participation more commonly experienced by underserved patients are addressed in the research design, such as physically locating study activities in community institutions, no‐cost transportation services for participants, after‐hour or weekend opportunities to complete study activities, and ensuring financial coverage for any incidental or secondary health expenditures resulting from study participation, a practice that should benefit from the recent expansion of Medicaid to cover routine clinical trial expenses.^44^, ^45^ At a minimum, researchers should ensure that data on race/ethnicity and social determinants of health is captured and reported to document existing data gaps in published cancer clinical trials.^46^ Many of the strategies endorsed by our participants, and those previously identified in the literature,^47^ require long‐term investment and broad structural changes to improve healthcare and research access, as well as critical outreach to historically underserved communities. Nevertheless, the commitment to redressing disparities in cancer research ultimately rests with individual researchers and institutions.

### 4.2 Strengths and limitations

To the best of our knowledge, this is the largest qualitative interview study conducted with Black women with a personal or family history of breast and ovarian cancer. Recall bias is a potential limitation, especially for the small number of participants with a more distant diagnosis. Family members also may not have full knowledge of their relatives' experience. We did not assess participants' baseline knowledge of clinical research; participants may have had differing or misconceptions of research activities. This cohort was more highly educated and privately insured than the national population on average; according to recent census data, 30.8% of Black women over age 25 have a bachelor's degree or higher, compared with 42.9% of non‐Hispanic White women.^48^ As educational attainment and health literacy are associated with research interest and participation,^23^ this may have influenced participants’ views. Barriers to research access are further magnified in lower socioeconomic populations; we are currently interviewing participants from a safety net clinic, whose experiences are likely to differ from the present cohort.

## 5 CONCLUSION

Our data suggest that lack of trust in the research community is not an insurmountable barrier to increasing enrollment of Black cancer patients. Researchers should implement the strategies outlined by our participants to begin to remediate systemic underinvestment in Black health and ensure equal access to advances in breast and ovarian cancer therapeutics.

## AUTHOR CONTRIBUTIONS

**Kirsten A Riggan:** Formal analysis (lead); investigation (equal); methodology (equal); writing – original draft (lead); writing – review and editing (equal). **Abigail Rousseau:** Formal analysis (equal); writing – review and editing (equal). **Michele Y. Halyard:** Conceptualization (equal); methodology (equal); writing – review and editing (equal). **Sarah E. James:** Writing – review and editing (equal). **Marion Kelly:** Conceptualization (equal); methodology (equal); writing – review and editing (equal). **Daphne Phillips:** Methodology (equal); writing – review and editing (equal). **Megan A Allyse:** Conceptualization (lead); formal analysis (equal); methodology (equal); writing – review and editing (equal).

## FUNDING INFORMATION

This project was supported by Mayo Clinic's Breast and Ovarian Specialized Program of Research Excellence (SPORE)/Women's Cancer Program Developmental Research Program award (Breast SPORE (P50 CA116201), the Ovarian SPORE (P50 CA136393), and Cancer Center Grant (P30 CA015083)). The funder had no involvement in the study design; in the collection, analysis, and interpretation of data; in the writing of the manuscript; and in the decision to submit the manuscript for publication.

## CONFLICT OF INTEREST

The authors declare no conflicts of interest.

## ETHICAL APPROVAL

All participants provided written informed consent and HIPAA authorization for their anonymous results to be used in this research. The study protocol was approved by Mayo Clinic's Institutional Review Board as minimal risk (#20–006851) and was performed in accordance with US Federal Policy for the Protection of Human Subjects.

## Supporting information


Appendix S1:

## Data Availability

The data that support the findings of this study are available from the corresponding author upon reasonable request.
