# Supplementary material for: “There's not enough studies”: Views of black breast and ovarian cancer patients on research participation
Source: Cancer Med. 2023 Jan 16;12(7):8767–76. doi: 10.1002/cam4.5622 (PMC10134334; doi:10.1002/cam4.5622)
Supplement: Supplementary file 1 — Appendix S1: [file CAM4-12-8767-s001.docx]

**Supplemental Materials**

Riggan KA, Rousseau A, Halyard M, James SE, Kelly M, Phillips D, Allyse MA. “There’s Not Enough Studies”: Views of Black Breast and Ovarian Cancer Patients on Research Participation. *Cancer Medicine*.

**Appendix S1. Interview Guide on Breast and Ovarian Cancer Experiences**

**Diagnosis and Treatment**

Can you tell me about your experience with BOC? [Note: the following questions are follow-ups depending upon the level of detail provided by the participant]

**Breast Cancer:** Do you know what type of breast cancer you had? Do you know the stage or how advanced the cancer was? For example, was it only in the breast or in the lymph nodes too? Did it spread beyond the breast and if so where?

**Ovarian Cancer:** Do you know the type of ovarian cancer you had? Do you know the stage or how advanced the cancer was? For example, had it spread outside the ovaries and if so where?

What age were you diagnosed?

Do you have a family history of BOC? If so, what family member? Do you know their age at diagnosis?

Did you have symptoms that prompted you to seek medical care? [e.g. changes in the Breast - lump, changes in breast shape or texture, nipple discharge; Ovarian – bloating, pelvic or abdominal pain, urinary/bowel symptoms]. About how long were you experiencing symptoms before you discussed them with a health care provider (HCP)?

How was your cancer diagnosed? [e.g. mammogram, pelvic exam, incidental finding].

What treatment options did your HCP discuss with you?

How soon after the diagnosis did you begin treatment?

Who did you discuss your treatment options with? [e.g. family, friends, support group, pastor/faith community]

What treatment(s) did you complete? [e.g. chemotherapy, hormonal therapy, radiation, lumpectomy, mastectomy – single or bilateral; partial or total hysterectomy, unilateral or bilateral salpingo-oophorectomy, omentectomy] Was this different from treatment options recommended by your provider?

Why did you choose that treatment option?

[If non-surgical approach chosen] Was surgery something you were willing to consider? Why or why not?

Did you have any fears or concerns about the treatment options for BOC?

Can you tell me about your insurance status at the time of your diagnosis/treatment?

Did your HCP discuss the possibility of participating in a study examining an experimental treatment (i.e. clinical trial) with you? Is this something you might have been interested in? Why or why not?

Did you undergo any sort of genetic testing before or after your cancer diagnosis? [e.g. BRCA1/2, Tumor DNA sequencing]

Did your HCP discuss a prognosis with you? Was this something that you wanted to know?

[If treatment is completed] Did your HCP recommend a plan to monitor for cancer return? What is your followup schedule?

**Experience with Medical System**

Do you feel that you received the support that you needed from your HCP(s) in terms of the diagnosis and treatment options?

Before your diagnosis, how comfortable did you feel going to the doctor? About how often would you have a health care appointment? Did you have a doctor you could trust?

How comfortable did you feel with your HCP [primary care provider and/or HCPs involved in cancer diagnosis/treatment]? What is their gender and race/ethnicity? Do you think their gender and/or race/ethnicity impacted your comfort level?

Do you feel more or less comfortable now after your diagnosis and/or treatment seeking medical care?

Do you speak with anyone about your thoughts/feelings/experiences with the medical system?

Would you say that there is a fear of seeking out medical care among the Black community? Fear of surgical interventions or certain procedures? [If yes:] What is the source of the fear? Is this something you discuss this with others?

Do you think there is a generational divide in how individuals experience/interact with their health care provider? Do you think the presence of an advocate [family member or third-party] would change this?

**Support During Cancer Experience**

What type of support [mental/emotional/spiritual/practical] did you need during your cancer journey?

Who did you turn to for support? [e.g. family, friends, pastor/faith community, support group]?

What sort of support was available to you?

What was the most helpful form of support to you?

What was the least helpful?

Did you have any support needs that were not met? How could these needs have been met?

What advice would you give to those supporting someone on their cancer journey?

What conversations should you avoid having with someone on journey through a breast or ovarian cancer diagnosis?

What does being a survivor mean to you?

Can you tell me about the long-term impact of your cancer experience? [effects of treatment, spiritual/mental/emotional/physical impact]. Cognitive burden and stress of cancer return? What you feel you might have lost?

**Cancer Screening and Testing**

Before you were diagnosed, what did you know about screening for early cancer detection? Did you undergo any type of routine screening? What types of screening [mammogram, self-breast exam, pelvic exam, pap smear, etc.]? How often?

Has anyone ever discussed cancer screening with you [HCP, family, friend(s), pastor, other BOC survivors]?

Is cancer screening something that you discuss with others? Can you tell me about those conversations?

How do these conversations take place? Where do they take place? [over dinner, who initiates the conversation] How often do they occur?

Are your family/friends/etc. open to these conversations?

What do you think is the best way(s) of introducing these topics to others?

[If no] Is there something that makes you hesitant to have these conversations? Other barriers?

Do you think it is difficult for people to acknowledge that cancer is something that might happen to them? Why or why not?

Have you heard of genetic testing for cancer risk before? Can you tell me what you have heard/know?

[If they haven’t heard of before] Just like you can pass on physical traits like eye color or hair color, you can also pass on risk of cancer to your children. Doctors can take a sample of blood, saliva, or a swab from the inside of your cheek to see if you have a change in certain genes that increase your risk of breast and ovarian cancer. The most widely known genes are BRCA1/2, but there are several other genes that can also increase your risk for cancer.

This test is often recommended to people who have a personal or family history of breast cancer, especially if diagnosed under the age of 50 or if the cancer was aggressive or recurrent.

Does this sound like something you might be interested in? Do you think this is something your family members would be interested in?

What do you think might be the benefits of genetic testing for cancer risk?

Do you have any particular concerns about genetic testing for cancer risk?

If asked or if someone made you aware of, would you be interested in participating in breast/ovarian cancer studies? Why or why not?

Do you have any particular concerns about participating in a study or clinical trial?

What do you think the benefits or outcomes of participating in a study might be? [e.g. benefit to you, benefit to science, etc.]

Do you think individuals from the Black community are less likely to participate in a research study or clinical trial? Why or why not?

**Concluding Questions**

If you could go back in time and give yourself advice about your cancer experience, what would you say?

If you had a magic wand and could change anything about screening or treatment for BOC what would that be?

Is there anything else you would like to share with me about your experience or your hopes for the future?

**Demographics**

Finally, I have a few demographic questions that will help us with our analysis.

What is your age?

How would you describe your overall health? [prompt: excellent, good, fair, poor]

What is your highest level of education?

What is your employment status? [prompt: working full-time, part-time, retired, unemployed, on disability]

What is your 9- or 5-digit zip code?

**Table S1. Sample Coding Schema on Research Participation**

| Code | Definition | Sample Quote |
| --- | --- | --- |
| Barriers | Discussion of barriers to research participation (e.g., transportation, not offered in their community, financial concerns, etc.) | *I think it’s not just because Black women don’t participate in these things. It’s not just ’cause we just don’t do it. I think it’s more complicated than that, and like I say, I think those barriers. When you think about the women that are usually eligible for clinical trials, those are usually younger women of childbearing age, possibly have children, are working, all those barriers, in addition, to the out-of-pocket costs ’cause that’s real.-*A47 |
| Dependent Upon Individual | Excerpts about how Black community is not a monolith in its views on research participation; interest in participation varies by individual. | *It’d be hard for me to speak for the whole Black community because most of the people that I associate with fall in a certain economic class. They’re a working class, people that see things from a more positive, progressive perspective that probably would say yes to participatin’ in a study. Then there are those that may be livin’ on the margin that may be not be willing to participate in a study. It just depends on their philosophical view, and where they are in their understanding of what study they may be able to do to assist or aid them?* -A31 |
| Historical Abuses | Discussion of Tuskegee Syphilis Trial, Henrietta Lacks, and other historical exploitative studies. May also include more recent events (e.g., hysterectomy of incarcerated individuals). | *Just not trusting, not trusting what the study is really for or intended. Black men, there was the syphilis case. It was literally injected into them. I can’t recall right now what they originally thought they were being—a placebo or whatever they were testing for but, yeah, were injected with syphilis. All of these things, it may not be common knowledge to everyone, but at least for the most part, our community is very aware of it. The word “study,” like, “This doctor wants to do this study,” is not an inviting phrase.* -A17 |
| Importance of | Excerpts on why it is important for Black individuals to participate in research (e.g., more tailored treatments, etc.) | *I want to help in educating the Black, African-American, Brown communities that more we know the better it can be for everyone for generations to come. It's important 'cause I knew some stats, but I've learned so much more going through this with my own experience. Yeah, there's no way if we don't open up, if we don't feel comfortable sharing our* *story, talking about our experiences, then we can't help that future. I know there are disparities in the way we survive this whole thing. I wanna help, however I can.* -A38 |
